# MBOAT7 expression is associated with disease progression in COVID-19 patients

**DOI:** 10.1007/s11033-023-09009-9

**Published:** 2024-01-06

**Authors:** Eman Radwan, Ahmed Abdelaziz, Manal A. M. Mandour, Abdel-Raheim M. A. Meki, Maha M. El-kholy, Marwan N. Mohamed

**Affiliations:** 1 Department of Medical Biochemistry, Faculty of Medicine, Assiut University, Assiut, 71515 Egypt; 2 Department of Biochemistry, Sphinx University, New Assiut City, Assiut 10 Egypt; 3 Department of Biochemistry, Faculty of Pharmacy, Assiut University, Assiut, 71515 Egypt; 4 Department of Chest diseases and Tuberculosis, Faculty of Medicine, Assiut University, Assiut, Egypt

**Keywords:** COVID-19, SARS-CoV-2, MBOAT7, Inflammation, Phospholipids

## Abstract

**Background and aim:**

The emergence of severe acute respiratory syndrome coronavirus 2 (SARS-CoV-2) in late 2019 caused a pandemic of acute respiratory disease, named coronavirus disease 2019 (COVID-19). COVID-19 became one of the most challenging health emergencies, hence the necessity to find different prognostic factors for disease progression, and severity. Membrane bound O-acyltransferase domain containing 7 (MBOAT7) demonstrates anti-inflammatory effects through acting as a fine-tune regulator of the amount of cellular free arachidonic acid. We aimed in this study to evaluate MBOAT7 expression in COVID-19 patients and to correlate it with disease severity and outcomes.

**Methods:**

This case-control study included 56 patients with confirmed SARS-CoV-2 diagnosis and 28 control subjects. Patients were further classified into moderate (n = 28) and severe (n = 28) cases. MBOAT7, tumor necrosis factor-α (TNF-α), and interleukin-1ß (IL-1ß) mRNA levels were evaluated in peripheral blood mononuclear cells (PBMC) samples isolated from patients and control subjects by real time quantitative polymerase chain reaction (RT-qPCR). In addition, circulating MBOAT7 protein levels were assayed by enzyme-linked immunosorbent assay (ELISA).

**Results:**

Significant lower levels of circulating MBOAT7 mRNA and protein were observed in COVID-19 patients compared to control subjects with severe COVID-19 cases showing significant lower levels compared to moderate cases. Moreover, severe cases showed a significant upregulation of TNF-α and IL-1ß mRNA. MBOAT7 mRNA and protein levels were significantly correlated with inflammatory markers (TNF-α, IL-1ß, C-reactive protein (CRP), and ferritin), liver enzymes, severity, and oxygen saturation levels.

**Conclusion:**

COVID-19 is associated with downregulation of MBAOT7, which correlates with disease severity.

## Introduction

The emergence of severe acute respiratory syndrome coronavirus 2 (SARS-CoV-2) in late 2019 caused a pandemic of acute respiratory disease, named ‘coronavirus disease 2019’ (COVID-19) [1, 2]. Multiple organs are affected by SARS-CoV-2 infection, due to multiple pathological mechanisms, including exaggerated immune responses triggered by the overproduction of proinflammatory cytokines, as tumor necrosis factor- α (TNF-α), interleukin-6 (IL-6), and interleukin-1ß (IL-1ß) that lead to the development of an uncontrolled inflammatory state known as the cytokine storm (CS) [3–7]. Moreover, COVID-19 mortality is directly correlated with the upregulation of cytokines [8].

Membrane-bound O-acyltransferases (MBOATs) superfamily, also known as lysophospholipid acyltransferases (LPLATs), comprises a number of genes involved in a variety of biological processes, such as embryogenesis, nutrient sensing, lipid biosynthesis, and membrane phospholipid remodeling [9].

Membrane bound O-acyltransferase domain containing 7 (MBOAT7) is well expressed by all major immune cell subsets [10]. It is primarily involved in the membrane phospholipid remodeling pathway known as the Lands’ cycle [11, 12]. In this process, phospholipase A_2_ (PLA_2_) and MBOAT7 carry out sequential deacylation and reacylation reactions consequently creating membrane fluidity and asymmetry [11, 12]. MBOAT7 plays a unique role in selectively diversifying the polyunsaturated fatty acid (PUFA) composition of phosphatidylinositol (PI) at the nucleophilic substitution 2 position through integrating free arachidonic acid (AA) in the form of arachidonoyl-CoA into lysophosphatidylinositol (LPI), releasing newly remodeled PI and limiting the availability of AA [13–17]. Thereby, the anti-inflammatory impact of MBOAT7 can be primarily explained by its ability to serve as a fine-tune regulator of the quantity of free AA, a known substrate for the synthesis of inflammatory lipid mediators as eicosanoids [17].

Severe COVID-19 cases were reported to be associated with aggravated liver injury [18, 19]. Previous studies reported that impairment in MBOAT7 functions promotes liver disease progression [20–26]. MBOAT7 was reported to negatively regulate Toll-like receptors (TLRs) signaling in both metabolic-associated fatty liver disease (MAFLD) and COVID-19 [27, 28]. Interestingly, polymorphism in the MBOAT7 gene was associated with severe liver injury in hospitalized COVID-19 patients [29].

To date, MBOAT7 has not been extensively investigated in COVID-19, hence in the present study, we aimed to evaluate MBOAT7 expression in COVID-19 patients and to correlate the levels of this anti-inflammatory enzyme with disease severity and outcomes.

## Subjects and methods

### Subjects

This case-control study included 84 subjects recruited between January 2021 and December 2021. The study included two groups: (Group 1) COVID-19 cases: included 56 patients aged more than 18 years admitted to the isolation unit of Assiut University Hospitals. All patients had a confirmed SARS-CoV-2 diagnosis by real-time quantitative polymerase chain reaction (RT-qPCR) testing of a nasopharyngeal swab specimen. (Group 2) Healthy control group: included 28 age and sex matched asymptomatic subjects with a confirmed negative SARS-CoV-2 by RT-qPCR testing of nasopharyngeal swab, no chest computed tomography (CT) scan abnormality and had no history of cancer, infection, or systemic disease. Subjects less than 18 years old, patients with chronic obstructive pulmonary disease, chronic kidney disease or liver cirrhosis were excluded from the study in addition to patients on dialysis, cancer patients, and pregnant women. A full medical history including demographic data and presenting symptoms was recorded. CT scan in addition to routine laboratory investigations including complete blood counts (CBC), liver and kidney function tests, serum C-reactive protein (CRP), serum ferritin, and serum D-dimer were performed for all patients.

COVID-19 patients were further classified into moderate (n = 28) and severe cases (n = 28). Moderate disease was defined by evidence of lower respiratory disease during clinical assessment or imaging and oxygen saturation measured by pulse oximetry (SpO2) ≥ 94% on room air at sea level. Severe disease was defined by SpO2 < 94% on room air at sea level, a ratio of arterial partial pressure of oxygen to fraction of inspired oxygen (PaO2/FiO2) < 300 mm Hg, a respiratory rate > 30 breaths/min, or lung infiltrates > 50% [30]. The present study procedures were approved by the Assiut Medical School Institutional Review Board, Assiut University, Egypt (IRB No. 17101694) and were performed in agreement with guidelines of the declaration of Helsinki. A written informed consent was obtained from all participants.

### Sample collection and processing

A volume of six milliliters of blood were collected in ethylene diamine tetraacetic acid (EDTA) containing tubes. Plasma was obtained by centrifugation of the blood at 2500 rpm for 10 min at 4 °C, then was aliquoted and stored at − 80 °C. The cell layer was used for fresh separation of peripheral blood mononuclear cells (PBMCs) by Ficoll-Hypaque density gradient centrifugation. Freshly obtained PBMCs were either directly processed for RNA extraction or were added to 800 µL TRIzol, snap frozen in liquid nitrogen and stored at − 80 °C till further use.

### Enzyme-linked immunosorbent assay

MBAOT7 levels in plasma were measured using a human MBOAT7 ELISA kit (Cat. No. #SG-15760, SinoGeneClon Co., Ltd, China) according to the manufacturer’s recommendations. All samples were assayed in duplicates and measured at a wavelength of 450 nm. The concentration of MBOAT7 was calculated based on the standard curve and expressed as pg/mL of plasma.

### RNA extraction and real-time qPCR

 RNA was extracted from PBMC samples using the RNeasy spin columns (Cat#74104, Qiagen, Germany). DNase treatment and negative controls were used to eliminate genomic DNA contamination. RNA purity and concentration were assessed by a Nanodrop spectrophotometer (Biotek, USA). 800 ng of RNA were reverse transcribed to complementary DNA (cDNA) using the high-capacity reverse transcription kit (Cat#4368814, Applied Biosystems, USA). qRT-PCR reactions were carried out using the Maxima SYBR® green/ROX RT-qPCR master mix kit (Cat#K0232, Thermo Fisher Scientific, USA). 20 µL reaction mixture composed of 10 µL Maxima SYBR® green/ROX RT-qPCR master mix, 6.4 µL nuclease-free water, 3µL of 2-times diluted cDNA template, and 0.3 µL of each forward and reverse primers (10 µM) was prepared. The mixture was then subjected to RT-qPCR in a StepOnePlus Real-Time PCR system (Applied Biosystems, USA). After an initial denaturation step for 5 min at 94 °C, a three-step cycling procedure (denaturation at 94 °C for 30 s, annealing at specific primer annealing temperature for 30 s, and extension at 72 °C for 60 s) was performed for 40 cycles. Expression data was normalized using GAPDH. Results were expressed as fold change by the 2^–ΔΔCT^ method. The primer sets used are shown in (Table 1). All kits and primers were used according to the manufacturer’s instructions.

**Table 1:** Primers used for RT-qPCR reaction

Gene	Sequence	Product length	Annealing temperature (°C)
GAPDH	Forward: 5-GACTAACCCTGCGCTCCTG-3 Reverse: 5-GCCCAATACGACCAAATCAG-3	136	49
MBOAT7	Forward: 5-CCTGCTCTCCTCTCACCTCT-3 Reverse: 5-AATCCAGGCCACGTAGAAGC-3	136	54
IL-1β	Forward: 5-ACAGATGAAGTGCTCCTTCCA-3 Reverse:5-GTCGGAGATTCGTAGCTGGAT-3	73	48
TNF-α	Forward: 5-TCTCTAATCAGCCCTCTGGCCCAGG-3 Reverse: 5-TACAACATGGGCTACAGGCTTGTCAC-3	78	52

*GAPDH* glyceraldeyde-3-phosphate dehydrogenase, *IL*-*1ß* interleukin-1 beta, *MBOAT7* membrane bound O-acyltransferase domain-containing 7, *TNF*-*α* tumor necrosis factor-α

### Statistical analysis

Statistical analysis was performed using Prism GraphPad Software 9.0.0. Variables were first tested to determine if data was parametric or non-parametric using the Shapiro–Wilk normality test. Statistical comparison between every two groups of continuous data was performed based on data distribution by either the independent t-test or Mann–Whitney *U* test. Categorical groups were statistically analyzed using the chi-square test. Analyses of correlation were performed based on data distribution by the Spearman’s or Pearson’s coefficients *U* tests. Continuous data were presented as mean ± SD, while categorical data as numbers and percentages (%). Receiver operating characteristic (ROC) analyses was performed by MedCalc® 20.104 software and used for evaluation of the area under curve (AUC), positive predictive values (PPV), negative predictive values (NPV), sensitivity and specificity. For all data, *p* value was considered significant if less than 0.05.

## Results

### Demographic and clinical characteristics of the studied patients

Regarding demographic data, the severe disease group had a higher age mean (63 ± 15 years) and included more male patients (64.28%) compared to the moderate group, despite no statistical significance. Also, the severe patient group had a higher incidence of diabetes and hypertension compared to the moderate group. Regarding symptomology, the most predominant symptom in the severe group was dyspnea, followed by cough and fever. The severe group had statistically significant dyspnea, higher respiratory rate, lower oxygen saturation, and higher mortality outcome compared to the moderate patient group (Table 2).

**Table 2:** Demographic and clinical characteristics of the study patients

Variable	Moderate COVID-19 (n = 28)	Severe COVID-19 (n = 28)	*p*-value
Age (mean ± SD)	60.89 ± 13.9	63 ± 15.15	0.8331
Gender (Male)	16 (57.14%)	18 (64.28%)	0.5842
Comorbidities			
Diabetes mellitus	9 (28.57%)	16 (57.14%)	**0.0308**
Hypertension	14 (50%)	18 (64.28%)	0.280
Clinical data			
Cough	11 (39.28%)	19 (67.85%)	**0.0321**
Dyspnea	6 (21.42%)	22 (78.57%)	**< 0.0001**
Chest pain	3 (10.71%)	1 (3.57%)	0.2994
Fever	12 (42.85%)	17 (60.71%)	0.1812
Headache	8 (28.57%)	3 (10.71%)	0.0926
Bone ache	4 (12.28%)	1 (3.57%)	0.1598
Fatigue	4 (12.28%)	6 (21.42%)	0.4853
Vomiting	4 (14.28%)	0 (0%)	**0.0379**
Diarrhea	4 (14.28%)	0 (0%)	**0.0379**
Loss of smell	3 (10.71%)	4 (12.28%)	0.6862
Respiratory rate (bpm)	26.39 ± 5.29	35.32 ± 6.8	**< 0.0001**
Oxygen saturation percentage (SpO2%)	90.12 ± 4.18	80.14 ± 13.38	**0.0027**
Outcome (non-survivors)	5 (17.56%)	20 (71.42%)	**< 0.0001**

*p* < 0.05 is considered significant (bold)

### Blood picture and chemistry

Severe cases showed no statistically significant difference from moderate cases regarding CBC. However, severe cases had lower mean hemoglobin concentration, mean platelets count, mean lymphocytes percentage, and higher mean neutrophils percentage. Liver function was significantly altered in severe cases compared to moderate cases and showed higher levels of alanine aminotransferase (ALT), aspartate aminotransferase (AST), alkaline phosphatase (ALP), direct bilirubin, whereas albumin was significantly lower in the severe group compared to moderate group. Also, the severe group had significant higher CRP and ferritin levels (Table 3).

**Table 3:** Laboratory investigations of the study patients

Variable	Moderate COVID-19 (n = 28)	Severe COVID-19 (n = 28)	*p*-value
HB	12.20 ± 2.09	11.15 ± 2.51	0.0944
PLT	274.46 ± 121.6	251.89 ± 140.63	0.5232
NEUT%	82.52 ± 15.48	85.99 ± 5.88	0.3724
Absolute LYMPH	1.46 ± 2.64	0.718 ± 0.315	0.0719
Albumin	34.71 ± 5.03	30.6 ± 5.2	**0.0040**
AST	45.11 ± 24.93	141.11 ± 94.52	**< 0.0001**
ALT	46.93 ± 24.38	132.07 ± 66.86	**< 0.0001**
ALP	78.18 ± 36.22	117.9 ± 68.62	**0.0073**
Total bilirubin	10.1 ± 8.24	19.63 ± 33.5	0.0538
Direct bilirubin	4.2 ± 4.3	10.38 ± 26.03	**0.0260**
Creatinine	108.5 ± 90.25	116.03 ± 77.17	0.5663
BUN	14.71 ± 13.7	12.8 ± 6.22	0.9579
CRP	83.95 ± 55.67	142.1 ± 87.84	**0.0263**
Ferritin	922.92 ± 1080.08	1860.28 ± 1479.86	**0.0003**
D-dimer	3.33 ± 2.92	5.33 ± 6.91	0.3262

*ALT* alanine transaminase, *ALP* alkaline phosphatase, *AST* aspartate transaminase, *BUN* blood urea nitrogen, *CRP* C-reactive protein, *HB* hemoglobin, *NEUT*% neutrophils percentage, *PLT* platelets count

*p* < 0.05 is considered significant (bold)

### Expression of MBOAT7

**Fig. 1:**
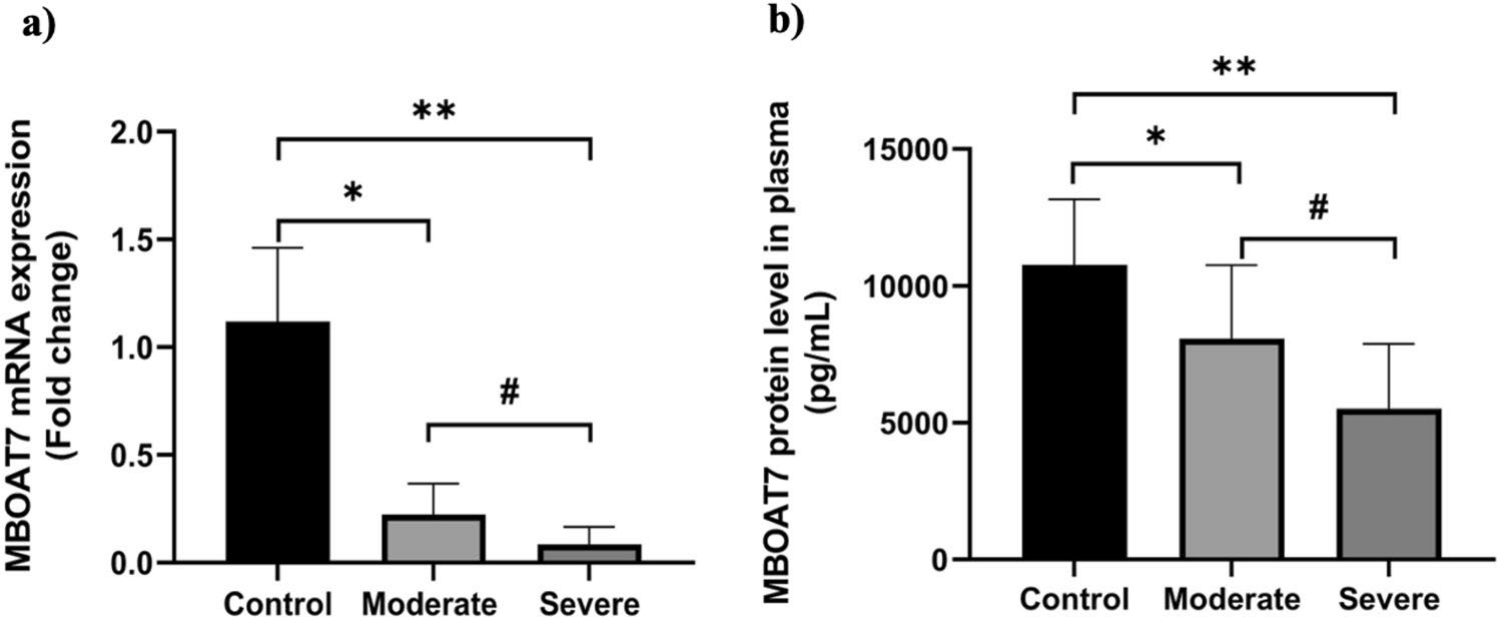
Expression of MBOAT7 in control subjects and COVID-19 patient groups. **a** MBOAT7 mRNA in PBMCs and **b** MBOAT7 protein in plasma. *p < 0.05 in control vs. moderate, **p < 0.05 in control vs. severe, and #*p* < 0.05 in moderate vs. severe

MBOAT7 mRNA and protein levels were significantly downregulated in COVID-19 patients group compared to controls (*p* < 0.0001). MBOAT7 mRNA was found to be significantly downregulated in severe cases compared to moderate cases (*p* = 0.0004). Moreover, plasma MBOAT7 protein level was significantly lower in severe cases compared to moderate cases (*p* = 0.0007) (Fig. 1).

### Expression of inflammatory cytokines

**Fig. 2:**
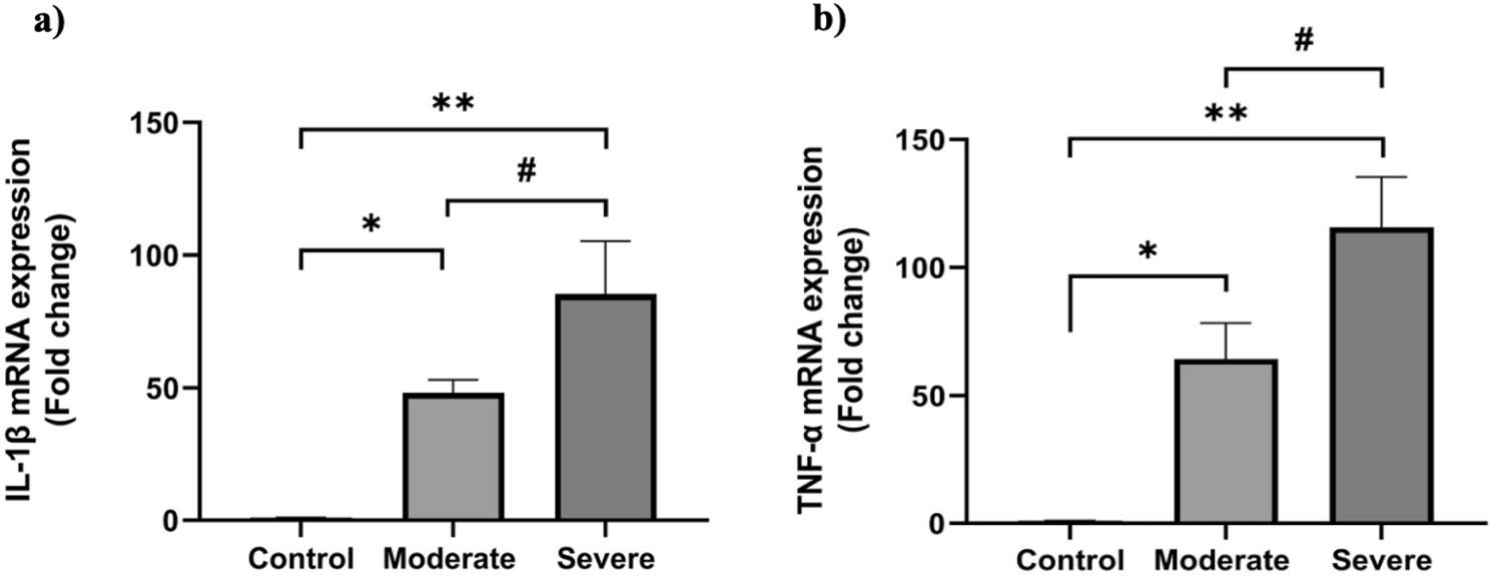
Expression of inflammatory cytokines in PBMCs of control subjects and COVID-19 patients **a** IL-1ß mRNA and **b** TNF-α mRNA. *p < 0.05 in control vs. moderate, **p < 0.05 in control vs. severe, and #*p* < 0.05 in moderate vs. severe cases

PBMCs isolated from COVID-19 patients (n = 56) showed significant upregulation of IL-1ß mRNA (Fig. 2a) and TNF-α mRNA in comparison to the control group (*p* < 0.0001) (Fig. 2b). Additionally, both genes were significantly upregulated in the severe COVID-19 group compared to the moderate group (*p* = 0.0004 for IL-1ß; *p* = 0.0004 for TNF-α) (Fig. 2).

### Evaluation of circulating MBOAT7 protein as markers for COVID-19 prediction

**Fig. 3:**
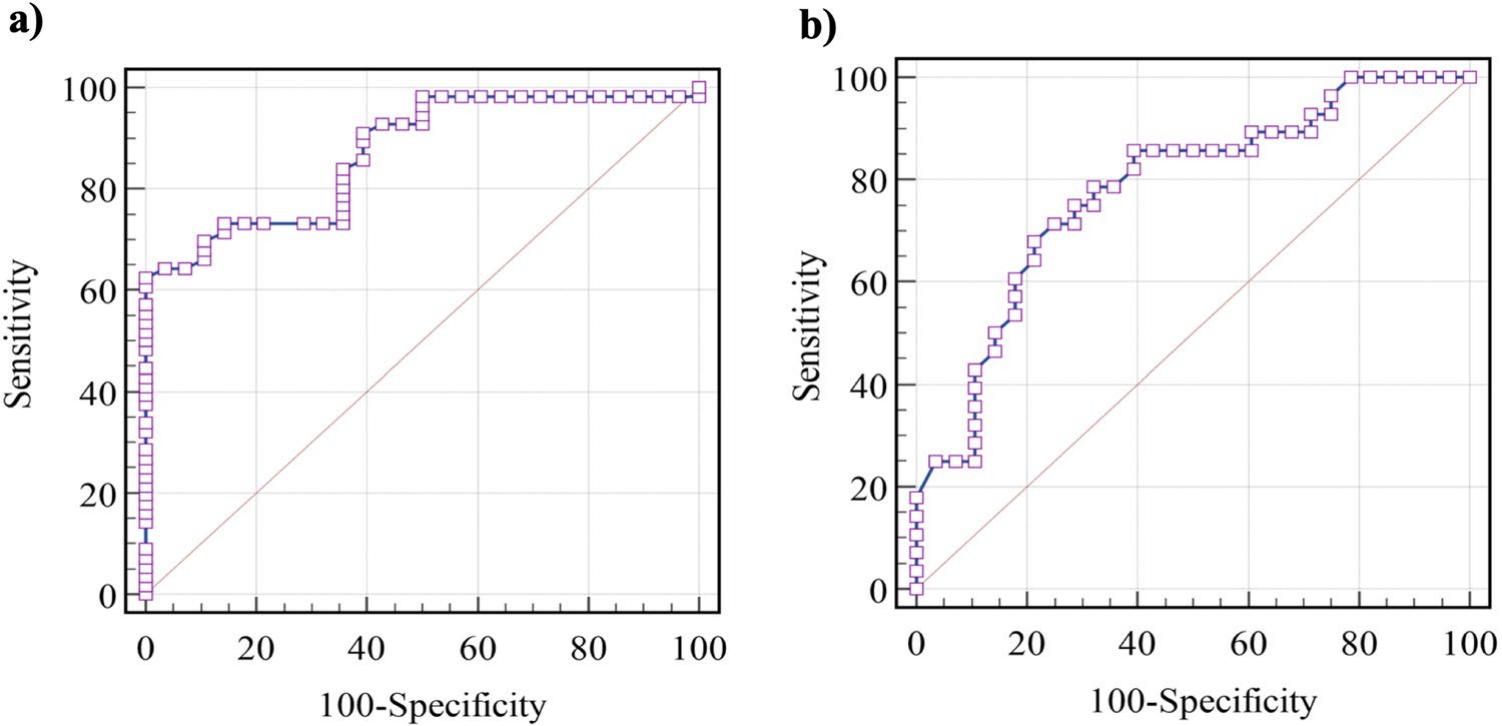
ROC curve analysis of circulating MBOAT7 protein level **a** in predicting COVID-19 and **b** in predicting severe COVID-19

The possible role of circulating plasma MBOAT7 protein level in predicting COVID-19 is illustrated in (Fig. 3a). MBAOT7 level was significantly altered in the patient group compared to the healthy controls with an AUC equal to 0.87 (*p* < 0.001) (Fig. 3a). Furthermore, the sensitivity, specificity, PPV and NPV were equal to 73.21%, 85.71%, 91.1%, and 61.5% respectively at a cut-off point of ≤ 8067.061 pg/mL. MBAOT7 protein level was good in predicting severe COVID-19, where the AUC was 0.778 (*p* < 0.001). The best cut-off point value was 6045.9 pg/mL. Accordingly, sensitivity, specificity, PPV and NPV were 71.43%%, 75%, 74.1% and 72.4%, respectively (Fig. 3b).

### Correlation analyses

In the present study, we examined the correlations between MBOAT7 mRNA, MBOAT7 protein, inflammatory cytokines (IL-1ß and TNF-α), CRP, Ferritin, D-dimer, CBC parameters, blood chemistry parameters, and SO2% in patients. As shown in Table 4, the results showed that MBOAT7 mRNA level in PBMCs was significantly positively correlated with MBOAT7 protein level in the plasma. In addition, both MBOAT7 gene expression and MBOAT7 protein level in the plasma were significantly positively correlated with SO2%. On the contrary, both MBOAT7 gene expression and its level in the plasma were found to be significantly negatively correlated with expression levels of inflammatory cytokines (IL-1ß and TNF-α), plasma level of acute phase reactants (CRP and ferritin), and liver enzymes (AST and ALT).

**Table 4:** Correlation analysis between MBOAT7 (mRNA and protein) levels and other relevant laboratory biomarkers in COVID-19 patients

Parameter	MBOAT7 mRNA expression (fold change)	MBOAT7 protein level (pg/mL)
MBOAT7 mRNA expression (fold change)		
MBOAT protein level (pg/mL)	r = 0.4177 ***p*** **= 0.0014**	
IL-1β expression (fold change)	r = − 0.3415 ***p*** **= 0.01**	r = − 0.4225 ***p*** **= 0.0012**
TNF-α expression (fold change)	r = − 0.4093 ***p*** **= 0.0017**	r = − 0.4565 ***p*** **= 0.0004**
CRP level (mg/L)	r = − 0.4581 ***p*** **= 0.0004**	r = − 0.3943 ***p*** **= 0.0026**
Ferritin (ng/mL)	r = − 0.2841 ***p*** **= 0.0338**	r = − 0.3070 ***p*** **= 0.0214**
HB (g/dL)	r = 0.3226 ***p*** **= 0.0153**	r = 0.03059 *p* = 0.8229
NEUT%	r = − 0.1930 p = 0.1541	r = − 0.07429 *p* = 0.5863
Absolute LYMPH	r = 0.4242 *p* = 0.1089	r = 0.0784 *p* = 0.5654
AST level (U/L)	r = − 0.3490 ***p*** **= 0.0084**	r = − 0.3251 ***p*** **= 0.0145**
ALT level (U/L)	r = − 0.4346 ***p*** **= 0.0008**	r = − 0.5027 ***p*** **= < 0.0001**
O2 saturation percentage (%)	r = 0.2881 ***p*** **= 0.0313**	r = 0.4339 ***p*** **= 0.0008**

*ALT* alanine transaminase, *AST* aspartate transaminase, *CRP* C-reactive protein, *HB* hemoglobin, *IL*-*1ß* interleukin-1 beta, *LYMPH* lymphocytes count; MBOAT7, membrane bound O-acyltransferase domain-containing 7, *NEUT*% neutrophils percentage, *TNF*-*α* tumor necrosis factor-α

Bold data represents statistically significant correlations

## Discussion

SARS-CoV-2 is a highly transmissible and pathogenic coronavirus that has been characterized by the development of the cytokine storm (CS) [31]. CS triggered by SARS-CoV‐2 infection is a central mediator for the lung injury and resulting acute respiratory disease found in cases of severe or critical COVID‐19 patients. CS also contributes to endothelial vascular dysfunction, multiorgan failure, alteration in iron homeostasis, and death [32]. Understanding the cellular, and molecular components that contribute to inflammation associated with COVID-19 is an important approach of great clinical significance and could substantially impact the public health.

Lipidomic studies have revealed that coronavirus modifies the lipid composition of infected cells [33]. Lands’ cycle is a series of decylation/reacylation reactions carried by PLA_2_ and MBOATs, resulting in remodeling of glycerophospholipids acyl chain [11, 12]. Previous studies addressed Lands’ cycle components and substrates association with COVID-19 [34–36]. PLA_2_ upregulation was proven essential for coronavirus replication and for the virus-induced inflammatory response [34]. Downregulation of PI and upregulation of AA were reported in the plasma of COVID-19 patients with AA correlated with severity of COVID-19 [36]. Other studies reported elevated LPI levels in severe COVID-19 cases [37, 38]. However, to date, the role of MBOAT7 in COVID-19 has not been evaluated.

MBOAT7 enzyme is a member of the MBOAT superfamily [39]. The main role of the MBOAT7 enzyme is membrane phospholipids remodeling through AA incorporation into LPI as a part of Lands’ cycle [17, 40]. MBOAT7 deficiency leads to dysregulated immune cell homeostasis, alterations in profiles of lipid mediators associated with AA redistribution, endoplasmic reticulum stress, mitochondrial dysfunction, and excessive release of cytokines [27].

In the present study, we found that MBOAT7 was significantly downregulated in COVID-19 patients compared to healthy controls. In addition, we found that circulating MBOAT7 plasma levels were lower in severe COVID-19 cases compared to moderate cases. ROC curve analyses showed that MBOAT7 could be used in predicting COVID-19, with good discriminative ability between severe and moderate disease. These data suggest the reliability of circulating MBOAT7 levels as a potential biomarker of COVID-19 disease progression and severity.

COVID-19 as a viral infection, is characterized by unique hyperinflammatory signatures across all types of immune cells, among which is the upregulation of IL-1β-, IL-6, and TNF-α-driven inflammatory responses, especially in severe cases [41]. In accordance, the results of the present study showed upregulation of both TNF-α and IL-1β cytokines in COVID-19 patients compared to control subjects. Moreover, both inflammatory cytokines were elevated in severe COVID-19 compared to moderate cases. Furthermore, CRP and ferritin differed significantly between moderate vs. severe COVID-19 cases. Interestingly, MBOAT7 mRNA and protein levels were significantly negatively correlated with levels of pro-inflammatory cytokines (IL-1ß and TNF-α) and inflammation markers (CRP and ferritin) emphasizing the role of MBOAT7 in regulating inflammation and CS development. These results could suggest MBAOT7 downregulation as one of the mechanisms through which SARS-CoV-2 manipulates the body inflammatory status [34].

It has been demonstrated that COVID-19 infection showed an augmented inflammatory response, leading to the CS which is considered the main factor associated with organ failure and death [42]. Multiple proinflammatory cytokines were found elevated in the sera of COVID-10 patients, among which are IL-1β, IL-6, and TNF-α [43], which correlated with COVID-19 severity and mortality [44, 45]. Also, systemic CRP and ferritin levels showed a significant positive association with severity and were independent predictors of survival in COVID-19 patients [46–48]. Elevated liver damage biomarkers together with respiratory infection were reported by Zhang et al. from a 82 death samples, indicating liver failure as is a key player in COVID-19 progression [49].

In this study, both MBOAT7 protein and MBOAT7 mRNA were significantly negatively correlated with liver function markers (AST and ALT), while significantly positively correlated with SO2%. This is in agreement with a previous study by Viitasalo et al., who found that MBOAT7 variation correlated with high circulating liver enzymes, mainly ALT levels and CRP concentrations [22]. This data might suggest MBOAT7 downregulation as an additional mechanism contributing to SARS-CoV-2 induced multi-organ damage and systemic inflammation.

Since studies identified PLA_2_ inhibitors as therapeutic targets to reduce COVID-19 mortality [34, 50], future research could address therapy that upregulates MBOAT7 activity as a complimentary strategy to inhibitors of PLA_2_ in order to restore normal Lands’ cycle homeostasis and minimize inflammation or CS.

In conclusion, the present study results demonstrated significant association of circulating MBOAT7 protein, MBOAT7 mRNA expression with the level of inflammation, severity, and outcome in COVID-19 patients.

## Limitations

The relatively small sample size represents the main limitation of this study. Larger samples are required to further confirm the results with inclusion of mild and critical COVID-19 patients. In addition, lack of follow up may affect the results, therefore, future research including follow up on patients and taking into consideration the effects of various therapy on circulating MBOAT7 levels is needed. Furthermore, the clinical course could be observed in more detail if analyses were performed at the onset of symptoms and not at admission only as demonstrated in this study.

## Data Availability

All related data and materials are available from the corresponding author upon request.
